# Opportunities and challenges for people-centered multi-hazard early warning systems: Perspectives from the Global South

**DOI:** 10.1016/j.isci.2025.112784

**Published:** 2025-06-06

**Authors:** Mirianna Budimir, Robert Šakić-Trogrlić, Cinthia Almeida, Miguel Arestegui, Orlando Chuquisengo Vásquez, Abel Cisneros, Monica Cuba Iriarte, Adama Dia, Leon Lizon, Giorgio Madueño, Alioune Ndiaye, Miluska Ordoñez Caldas, Tamanna Rahman, Bikram RanaTharu, Alpha Sall, Dharam Uprety, Chris Anderson, Colin McQuistan

## Main text

(iScience *28*, 112353; May 16, 2025)

During typsetting, the supplier tagged the authors’ first names as surnames and vice versa. This has now been updated online. We apologize for this oversight.

